# Electrochemical synthesis of high entropy nanoparticles and the exploration of the Pd–Ag–Au composition space for the oxygen reduction reaction†

**DOI:** 10.1039/d5fd00095e

**Published:** 2025-07-08

**Authors:** Menglong Liu, Divyansh Gautam, Christian M. Clausen, Ahmad Tirmidzi, Gustav K. H. Wiberg, Jan Rossmeisl, Matthias Arenz

**Affiliations:** a Department of Chemistry, Biochemistry and Pharmaceutical Sciences, University of Bern Bern 3012 Switzerland matthias.arenz@unibe.ch; b Center for High Entropy Alloy Catalysis (CHEAC), Department of Chemistry, University of Copenhagen København Ø 2100 Denmark

## Abstract

Multi-metallic alloys such as high entropy alloys (HEAs) span an extensive compositional space, potentially offering materials with enhanced activity and stability for various catalytic reactions. However, experimentally identifying the optimal composition within this vast compositional space poses significant challenges. In this study, we present a medium-throughput approach to screen the composition–activity correlation of electrodeposited multi-metallic and HEA nanoparticles. We apply the approach for exploring the Pd–Ag–Au composition subspace for the alkaline Oxygen Reduction Reaction (ORR). The Pd–Ag–Au alloy nanoparticles were synthesized electrochemically, characterized and evaluated for the ORR using a rotating disk electrode (RDE) setup. From 107 individual measurements, a composition–activity correlation model was constructed using Gaussian Process Regression (GPR), pinpointing the optimal composition around Pd_85_Ag_1_Au_14_. The experimental results are then compared to theoretical predictions based on the well-established descriptor approach utilizing density functional theory (DFT) calculations. While some discrepancies exist, the experimental DFT-derived models show partial overlap, validating the utility of computational screening for multi-metallic systems. This work provides valuable insights for the efficient screening of multi-metallic catalysts for catalytic applications and exemplifies advanced pathways on how to compare and analyze experimental data to simulations based on well-defined hypotheses.

## Abstract 2

Medium-throughput exploration of Pd–Ag–Au compositional space for ORR and the comparison between experimental and DFT calculation models.

## Graphical abstract

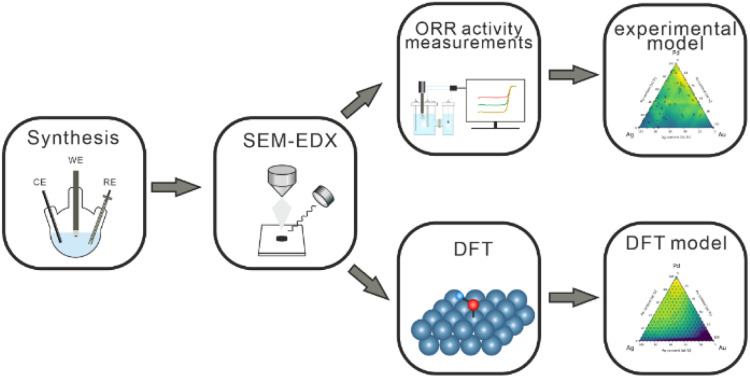

## Introduction

Due to the energy crisis and environmental problems caused by the massive use of fossil fuels, the demand for clean and sustainable energy conversion technologies has kept growing in the past decades.^1^ Fuel cells, particularly proton exchange membrane fuel cells (PEMFC) and alkaline fuel cells (AFC), have emerged as one of the promising technologies for reducing greenhouse gas emissions and minimizing reliance on fossil fuels.^2,3^ These devices efficiently convert chemical energy (*e.g.* H_2_) directly into electricity through electrochemical reactions and have the advantages of higher energy efficiency and lower emissions compared to fossil fuel-based power plants. Their versatility allows applications such as hydrogen fuel cell powered (heavy duty) vehicles and energy storage devices. Among the reactions in fuel cells, the oxygen reduction reaction (ORR) at the cathode is critical for the overall performance due to the relatively sluggish reaction kinetics (4 e^−^ process).^4,5^ Therefore, developing catalytic active, stable and cost-effective electrocatalysts for the ORR is essential for advancing fuel cell technology.

The catalytic performance of ORR catalysts is highly dependent on the electrolyte environment, with acidic and alkaline conditions presenting distinct challenges and opportunities. In acidic media (*e.g.* 0.1 M H_2_SO_4_ or HClO_4_), Pt-based catalysts are the benchmark due to their exceptional activity and stability under such harsh conditions.^6,7^ However, the high cost and scarcity of Pt, as well as its susceptibility to CO poisoning, have driven research into alternative options, including Pt alloys and non-precious metal catalysts. In addition, an acidic environment poses the challenge of catalyst stability. In contrast, alkaline electrolytes offer a more favorable environment for non-precious metal catalysts, such as transition metal oxides (*e.g.*, MnO_2_, Co_3_O_4_), N-doped carbon materials, and single-atom catalysts (Fe–N–C and Co–N–C), which are reported to exhibit enhanced stability and comparable activity to Pt in alkaline media.^8–13^ However, the activity of these catalysts in alkaline media is still not comparable to that of Pt catalysts in acidic media. Catalyst development for the ORR therefore remains one of the main challenges for the widespread application of fuel cell technology.

Recently, high-entropy alloys (HEAs) have emerged as a promising platform to screen electrocatalysts. HEAs, which consist of five or more principal elements in near-equimolar ratios, exhibit high configurational entropy, lattice distortion, potentially leading to synergistic effects among multiple elements.^14,15^ In literature, unique structural and electronic properties are reported that lead to improved material properties such as corrosion resistance and enhanced catalytic activity.^16–18^ For example, FeCoNiCrMn/C has been reported to exhibit competitive ORR performance, rivalling Pt-based catalysts in alkaline media.^19^ In addition, these HEA characteristics enable tunable active sites by varying their composition. The compositional flexibility of HEAs – and lower dimensional (fewer components) subspaces – allows for systematic screening studies, not only identifying compositions with improved activity and durability but also providing a test ground for comparing theoretical and experimental results in unprecedented detail.^20,21^ While the approach has been established for thin-film HEA libraries,^22,23^ most experimental HEA research investigating nanoparticles only studies a limited number of compositions and the vast compositional space of HEAs (∼10^5^ possibilities for 5 elements assuming a compositional discretization of 1%) remains underexplored. The traditional one-at-a-time synthesis of nanoparticles fails to exploit their full potential, and despite recent progress, few systematic studies exist to explore HEA compositional spaces efficiently for nanoparticles. Current methods lack throughput or theoretical integration, leaving >99% of combinations untested.

Here, we bridge this gap by demonstrating a medium-throughput approach to map the ORR activity across Pd–Ag–Au trimetallic compositions. Building on our previous work,^24^ where we demonstrated the synthesis of HEA nanoparticles *via* electrochemical deposition, we employ a similar technique to directly deposit Pd–Ag–Au nanoparticles onto glassy carbon (GC) RDE tips with tunable compositions by varying the metal precursor ratios. The ORR activity of the electro-deposited Pd–Ag–Au nanoparticles is then evaluated in a separate cell with an RDE setup. Composition and particle coverage are evaluated by scanning electron microcopy (SEM) and energy dispersive X-ray spectroscopy (EDX). The experimental results are processed with Gaussian Process Regression (GPR) to build a model correlating composition and ORR activity, which is then compared with a model derived from DFT calculation. It is demonstrated that the experimental data align with DFT-calculated activity trends, with the optimal zone partially overlapping, thus validating computational screening for multi-metallic systems. This work establishes a template for accelerated HEA discovery, combining efficient experimentation with theoretical guidance.

## Experimental part

### Chemicals and gases

The following chemicals were employed for the electrochemical deposition of the catalysts and subsequent characterization: sodium chloride (NaCl, 99.99% Suprapur, Sigma-Aldrich), sodium sulfate (Na_2_SO_4_, 99.99% Suprapur, Sigma-Aldrich), potassium hydroxide (KOH, 99.99% trace metals basis, Sigma-Aldrich), ammonium tetrachloropalladate(ii) ((NH_4_)_2_PdCl_4_, 99.995% trace metals basis, Sigma-Aldrich), hydrogen tetrachloroaurate(iii) trihydrate (HAuCl_4_·3H_2_O, 99.9% trace metals basis, Sigma-Aldrich), sodium aurothiosulfate (Na_3_Au(S_2_O_3_)_2_·2H_2_O, 99.9% metals basis, Alfa Aesar), potassium dicyanoaurate(i) (KAu(CN)_2_, 99.95% trace metals basis, Sigma-Aldrich), silver nitrate (AgNO_3_, 99.995% metals basis, Alfa Aesar); ammonium hydroxide solution (ACS reagent, 28–30% NH_3_ basis). Ultrapure water (resistivity >18.2 MΩ cm, total organic carbon (TOC) <5 ppb) obtained from a Milli-Q system (Millipore) was used for acid/base dilutions, aqueous solution preparation, and electrochemical cell cleaning. High purity gases including argon (Ar, 99.999%), hydrogen (H_2_, 99.999%), and oxygen (O_2_, 99.999%), supplied by Air Liquide, were used for electrochemical measurements.

### Electrochemical setup and deposition protocols

Electrochemical deposition was conducted in a three-neck flask equipped with a three-electrode system. A glassy carbon RDE with a diameter of 5 mm (geometric surface area: 0.196 cm^2^) served as the working electrode (WE). A GC rod (5 mm diameter) was used as the counter electrode (CE), and a 3 M Ag/AgCl electrode served as the reference electrode (RE). All electrochemical experiments were carried out using an Eci-210 potentiostat from Nordic Electrochemistry ApS. Three distinct protocols were employed for the deposition of Pd–Ag–Au nanoparticles: galvanostatic, potentiostatic, and pulsed deposition.

For both the galvanostatic and potentiostatic protocols, a total charge of approximately −3.4 mC was applied. In the pulsed protocol, the potential was initially held at 0.5 V *vs.* Ag/AgCl for 120 s. During this period, the WE was inserted, and the resistance between the WE and RE (∼25 Ω) was reduced to ∼5 Ω using the potentiostat’s analog positive feedback scheme. The potential was then changed to −0.7 V *vs.* Ag/AgCl (*E*_N_) to initiate nucleation. Subsequently, it was alternated between a rest potential (*E*_R_) of 0 V *vs.* Ag/AgCl and a deposition potential (*E*_D_) of −0.65 V *vs.* Ag/AgCl, with each held for 0.05 s with a deposition time of around 60 s. For a typical deposition procedure, 11.5 mL of 0.1 M Na_2_SO_4_ was added to the flask and purged with Ar to remove dissolved gases. Subsequently, 20 μL of ammonium hydroxide was added, followed by the sequential addition of Ag, Pd, and Au precursor solutions. The total metal precursor concentration in the electrolyte was kept below 0.5 mmol L^−1^. Ammonium hydroxide was included to form soluble [Ag(NH_3_)_2_]^+^ complexes, thereby preventing AgCl precipitation. The three electrodes were then assembled as shown in Fig. S1,† and one of the deposition protocols was applied to deposit nanoparticles onto the GC RDE surface. After deposition, the RDE tip was thoroughly rinsed with Milli-Q water and dried before further characterization or ORR measurements.

### Varying the composition of electrodeposited nanoparticles

**Fig. 1:**
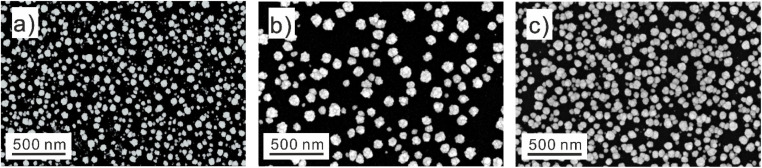
Representative SEM images of the nanoparticles obtained *via* the different electrochemical deposition methods. (a) Potentiostatic method with a potential of −0.6 V *vs.* Ag/AgCl; (b) galvanostatic method with a current of −70 μA; (c) pulsed protocol. The current/potential details of the deposition are shown in Fig. S3 and S4.†

Pd–Ag–Au nanoparticles with varying compositions were deposited using the three protocols (Fig. 1 shows representative SEM images). Most depositions were carried out using the pulsed protocol. By adjusting the concentrations of metal precursors in the electrolyte, we achieved compositional variation. For a few specific compositions, potentiostatic or galvanostatic methods were used. Although particle size and coverage could be fine-tuned by altering potential, current, or precursor concentration (Fig. S2† shows an example), we did not attempt to enforce a uniform particle size across all samples. Instead, we maintained a general size range of 50–100 nm, which is above the threshold where typically particle size effects (<10 nm) significantly impact the electrochemically active surface area (ECSA) specific ORR activity.^25,26^

### Scanning electron microscopy (SEM) and energy dispersive X-ray spectroscopy (EDX)

After thorough cleaning and drying, the RDE tips were mounted onto a custom SEM stage for SEM and EDX analysis using a Zeiss Gemini 450 SEM equipped with an InLens secondary electron detector. Imaging was performed at 5 kV acceleration voltage, 100 pA current, and a working distance of ∼5 mm.

For compositional analysis, EDX measurements were taken at 5 kV, 150 pA, and 8.5 mm working distance. Spectra were processed using Aztec 4.2 software to determine the elemental composition of the nanoparticles.

### ORR activity measurements

The ORR activity of the Pd–Ag–Au nanoparticles was evaluated using an RDE setup in a Teflon cell.^27^ The RDE tip served as the WE, a gold wire as the CE, and a leakless Ag/AgCl electrode as the RE. The RE potential was calibrated daily against a reversible hydrogen electrode (RHE). All ORR measurements were performed in freshly prepared 0.1 M KOH. A macro script developed using EC4DAQ automated the gas purging, RDE rotation and recording of the electrochemical data. Initially, the electrolyte was purged with Ar (200 mL min^−1^, 20 min), followed by recording cyclic voltammograms (CVs) at 25, 50, 100, and 250 mV s^−1^ (three cycles each) within a potential range of 0 to 1.1 V_RHE_ under Ar atmosphere. Subsequently, the solution was purged with O_2_ (same conditions; 200 mL min^−1^, 20 min), and CVs were recorded at rotation rates of 0, 100, 400, 900, 1600, 2500, 3600, and 4900 rpm (three cycles per rotation rate) with a scan rate of 50 mV s^−1^ within a potential range of 0 to 1.1 V_RHE_ under O_2_ atmosphere. During the measurements, the resistance between the WE and RE (∼25 Ω) was compensated to approximately 4 Ω using the potentiostat’s analog positive feedback scheme.

### Gaussian process regression (GPR) model

The GPR model was trained using compositions obtained from EDX as input features (*x*), where the sum of all elemental components equals one, and the target variable (*y*) was the ORR activity, which was normalized prior to modelling. The GPR model was implemented using the scikit-learn library.^28^ The kernel employed was a Pairwise Kernel (gamma = 1.00, gamma_bounds = (0.01, 1e5), metric = ‘laplacian’).

### Density functional theory calculations and simulated activities

The DFT calculations performed as part of this project used the revised Perdew–Burke–Ernzerhof (RPBE) exchange–correlation functional^29^ implemented in the GPAW code.^30,31^ All surfaces were modelled with ASE^32^ as 3 × 3 × 5 atom-sized fcc(111) slabs with lateral periodic boundary conditions and a vacuum of 10.0 Å added above and below the slab. Each slab had the *x* and *y* dimensions of the unit cell scaled to the weighted mean lattice constant of the elements found in the surface layer due to considerations on strains described in a previously published study.^33^ The atoms in the two bottom layers were held fixed during the relaxations, and the structures were then optimized towards a maximum force criterion of 0.1 eV Å^−1^. The wave functions were expanded in plane waves with an energy cutoff set to 400 eV, and the Brillouin zone was sampled with a Monkhorst–Pack grid^34^ of 4 × 4 × 1 *k*-points. Molecular gas phase references were used to calculate the adsorption energy as:

1 Δ*E*^DFT^_*ads_ = Δ*E*^DFT^_slab+ads_ − Δ*E*^DFT^_slab_ − Δ*E*^DFT^_ads_

with Δ*E*^DFT^_slab+ads_ and Δ*E*^DFT^_slab_ being the total energy of the slab with and without adsorbate, respectively. Δ*E*^DFT^_ads_ is the total energy of the adsorbate calculated from the molecular references in vacuum. Specifically, this equates to 
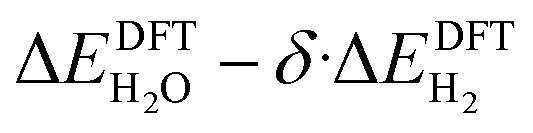
 with *δ* being 0.5 and 1.0 for *OH and *O.

The simulation of catalytic activities was achieved by applying a well-known kinetic activity model, which is exponentially dependent on the adsorption energies of *OH and *O as descriptors for ORR activity. Our assumption is that the catalytic process occurs on a stochastically composed surface characteristic of high-entropy materials greatly complicates these simulations as the adsorption energies are not a few discrete values but rather complex distributions due to the combinatorial explosion of unique binding sites on the surface. To obtain the adsorption energy distribution of a particular alloy composition, we employ an earlier published procedure of creating a surrogate surface to emulate a 96 × 96 atom-sized fcc111 surface to achieve statistically relevant sampling of the binding sites.^35^

Running structure optimizations with density functional theory on tens of thousands of binding site geometries to estimate the catalytic activity of a single alloy in a continuous composition space is obviously infeasible to the point of impossibility. Therefore, we employ machine learning-based models to infer the adsorption energy of each binding site so we can screen a catalyst composition in the order of minutes. Our inference model of choice is the EquiformerV2-31M (eqV2-31M) model^36^ which is pre-trained on the OC20 dataset.^37^ It has previously been shown that fine-tuning this model on HEA systems has proven effective for direct inference of *OH and *O adsorption energies in a so-called Initial-Structure-to-Relaxed-Energy (IS2RE) procedure, without the need of a time-costly geometry optimization.^38^

For this study, we have used a version of eqV2-31M which we have fine-tuned on a large dataset of DFT calculations that spans solid–solution HEAs in a composition space spanned by 12 different elements and 9 different adsorbates, including Ag, Au, and Pd as constituent elements and *OH and *O as adsorbed species. This dataset will be published in a separate upcoming publication, however ESI† for this publication holds the model checkpoint and dedicated test sets on Pd–Ag–Au and the binary sub-alloys, Ag–Au, Pd–Ag, and Pd–Au, to document the model performance. As seen in Fig. S5–S8,† the model achieves extremely low mean absolute errors across the test sets ranging from 0.015 to 0.034 eV for *OH and between 0.029 and 0.043 eV for *O.

By applying this model, we can obtain the gross *OH and *O adsorption energy distributions for any alloy in the Pd–Ag–Au composition space. Subsequently masking the binding sites deemed unavailable for adsorption, due to inter-adsorbate interactions and site-blocking, we get the net adsorption energy distributions.^35^ These are the descriptors that constitute the input to the expression for average kinetic current, *j*_k_, (measured in arbitrary units) calculated as:2
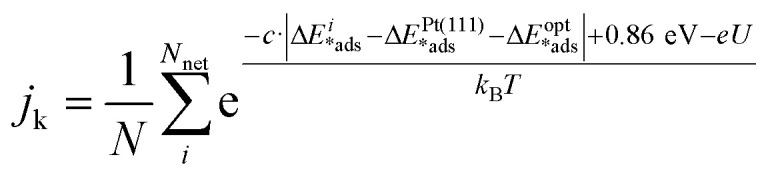
*c* and Δ*E*^opt^_*ads_ are set to 1.0 and 0.1 eV for *OH, with the same parameters being 0.5 and 0.2 eV for *O. Δ*E*^i^_*ads_ is the adsorption energy of binding site *i* and *T* was set to 298.15 K. *N* and *N*_net_ are the total number of surface sites and the number of occupied sites, respectively.

### Data analysis

**Fig. 2:**
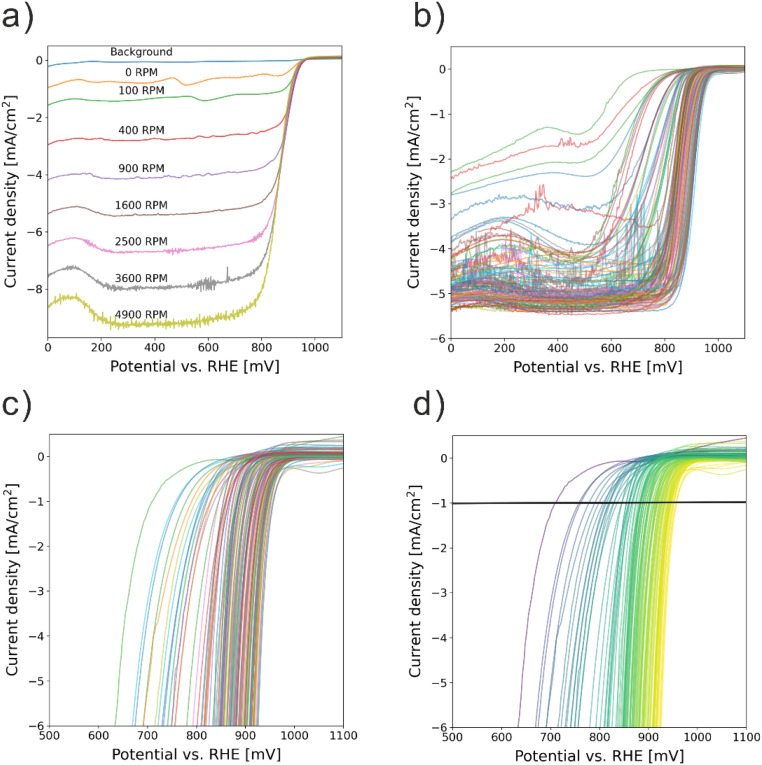
(a) Positive going LSV curves of a Pd_100_ sample at different RDE rotation rate; (b) positive going LSV curves recorded at 1600 rpm of all the measured 107 samples; (c) kinetic current densities normalized to the individual coverage of the 107 samples; (d) color-coded results of the normalized kinetic current densities from (c) indicating the “onset” potential at which a specific current density of −1 mA cm^−2^ was recorded.

Fig. 2a shows LSVs from a typical ORR activity test. The kinetic current density (*j*_k_) was extracted from the positive going scan recorded under O_2_ atmosphere (1600 rpm, 50 mV s^−1^), with the respective scan recorded under Ar atmosphere (0 rpm, 50 mV s^−1^) used for background correction. The Koutecký–Levich equation:
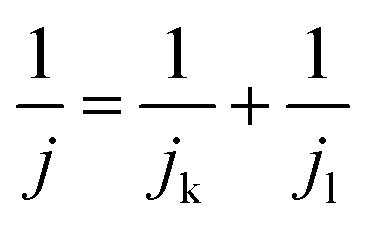
was applied to derive *j*_k_, where *j*_l_ is the diffusion-limited current density and *j* is the recorded current density.

Standard methods (*e.g.*, CV, CO stripping) to determine the ECSA of ORR catalysts risk altering the surface composition of multi-component nanoparticles. Furthermore, no precise standard stripping charges exist. Thus, we approximate nanoparticles as hemispheres. The kinetic current was then normalized to the nanoparticle-covered area on the GC electrode, determined from SEM image analysis. For this a Python script^39^ inverted (converting nanoparticles from white to black and the background from black to white) and enhanced the contrast of the SEM images. Then the coverage was calculated by determining the percentage of dark pixels (representing nanoparticles) relative to the total numbers of pixels in the image, with one example shown in Fig. S9.† The thus normalized kinetic currents were used to identify the WE potential at which −1 mA cm^−2^ was reached, from which the composition–activity contour plot was generated.

## Results and discussion

As outlined in the introduction, this study aims to systematically compare experimental ORR data with DFT-derived models to investigate the composition–activity relationship of Pd–Ag–Au trimetallic nanoparticles. In electrocatalysis, DFT is frequently used to support claims that specific compositions or structures exhibit enhanced catalytic activity. The core hypothesis is that the ORR activity – expressed in arbitrary units – can be predicted from the binding energies of key intermediates (*e.g.*, adsorbed OH_ad_), as discussed in the experimental section. Such “experiment–theory” comparisons often rely on a limited number of data points, either from a single experimental campaign or aggregated from different literature sources. Additionally, the calculated catalyst structures typically diverge from those actually tested. The resulting comparisons are often visualized using logarithmic volcano plots based on Sabatier’s principle, but these can feature significant outliers – particularly for low-performing catalysts that deviate by orders of magnitude from the trend. Systematic, large-scale comparisons using consistent experimental datasets remain rare. To address this, we implemented a medium-throughput experimental strategy to explore the Pd–Ag–Au compositional space. This approach, combined with GPR, enabled us to derive an experimental composition–activity model for comparison with DFT predictions.

### Exploration of the Pd–Ag–Au compositional space

A targeted sampling strategy was employed to efficiently map the compositional space of Pd–Ag–Au nanoparticles. Unlike conventional high-throughput methods, which require hundreds of uniformly distributed samples, our approach prioritized regions predicted to exhibit high ORR activity (*e.g.*, Pd-rich zones), reducing the number of required samples. We began by synthesizing Pd_90_Ag_10_ and Pd_90_Au_10_ nanoparticles, focusing on predicted optimal compositions. Sampling was then extended along the Pd–Ag and Pd–Au binary edges, leveraging the greater control afforded by electrodeposition of bimetallic systems. The ORR activities of monometallic Pd, Ag, and Au nanoparticles were also evaluated. To complete the ternary map, we attempted to construct a compositional grid using 15% increments. The Ag–Au binary edge was minimally sampled due to the known poor ORR activity of both pure Ag and Au.

**Fig. 3:**
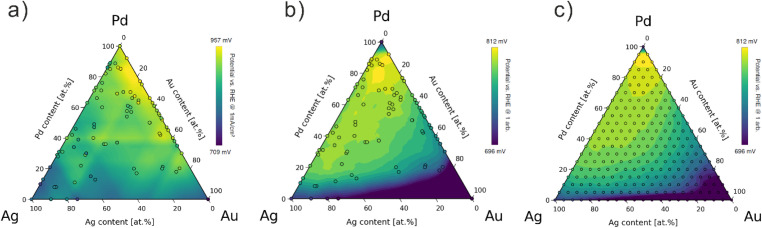
Simplexes representing the Pd–Ag–Au composition space with the sampled compositions overlaid as individual points with (a) the experimentally measured potentials at which the kinetic ORR current density reaches −1 mA cm^−2^; (b) the corresponding simulated potentials based on DFT calculations; (c) a finer DFT “grid model” using 5% composition steps. The background heatmap is obtained from fitting a GPR model to the data points.

In total, 107 samples (including replicates) were synthesized, characterized, and tested. Details of synthesis parameters (method, electrolyte composition, deposition time) are provided in the ESI Excel file.† LSVs for all samples are shown in Fig. 2b, from which the kinetic current densities were extracted and normalized by nanoparticle coverage, see Fig. 2c. For the contour plot, the potential at −1 mA cm^−2^ (normalized current density) was extracted for each sample and visualized on a color scale (Fig. 2d). GPR was then used to construct the experimental composition–activity model as shown in Fig. 3a (the accuracy of the GPR model is shown in Fig. S10†), correlating each metal ratio in the Pd–Ag–Au composition space with an ORR activity. We refer to this as the “experimental model”. The full dataset, including determined elemental compositions, surface coverages, and determined ORR “onset” potentials (potential at which a normalized current density of −1 mA cm^−2^ was reached) are also available in the ESI Excel file.† All raw data and python scripts to process the data are published in Zenodo.^40^

Analyzing the data obtained, it should be noted that the RDE setup provides several benefits for assessing the ORR activity of nanoparticle-based catalysts. Rotation creates a well-defined diffusion layer, enabling an accurate extraction of the intrinsic kinetic activity using the Koutecký–Levich equation and minimizing artifacts such as local pH gradients. The expected “shape” in the polarization curves is well established and the theoretical diffusion limited currents are known. In our overall data set, some potential outliers were identified while possible errors originating from the RDE setup have been excluded by showcasing normal distributed activity values when repeating the complete synthesis and testing procedure for a selected composition more than ten times (Fig. S11†).

The potential outliers may stem from various sources such as incomplete four-electron ORR, uneven nanoparticle deposition (leading to inaccurate coverage estimates), contamination, or O_2_ undersaturation. These outliers in principle can be identified for example by incomplete diffusion-limited plateaus or non-linear behavior in Koutecký–Levich plots. While 31 samples could have been flagged as potential outliers, it was noted that excluding them in the GPR model would have had minimal impact on the final experimental model (no comparison shown here but at the beginning of the measurement campaign such potential outliers had a significant influence on the derived experimental model), affirming the robustness of our data set. Thus, all measurements were retained for analysis and no potential outlier was excluded. The experimental model and the accuracy excluding the potential outliers are shown in Fig. S12 and S13.†

### Comparison between experimental model and DFT model

The experimental model (Fig. 3a) indicates that high Pd content with small amounts of Ag and Au gives the best ORR activity. The optimal compositions lie between Pd_85_Au_15_ and Pd_90_Ag_5_Au_5_, with maximum activity of higher than 957 mV_RHE_ at normalized current density of −1 mA cm^−2^. Monometallic Pd approaches this optimal activity (940 mV_RHE_), while Ag- and Au-rich compositions exhibit significantly lower performance (∼800 mV_RHE_). Along the Pd–Ag edge, the ORR activity decreases near monotonically from Pd_100_Ag_0_ to Pd_0_Ag_100_, whereas the Pd–Au edge displays a maximum between Pd_90_Au_10_ and Pd_70_Au_3_, then slowly declines to ∼900 mV_RHE_ at Pd_20_Au_80_. The Ag–Au edge shows the lowest ORR activity overall.

For comparison, a respective contour map was constructed using DFT-calculated binding energies (Fig. 3b), using the same composition set as in the experimental data. In addition, a finer “grid model” using 5% composition steps is shown in Fig. 3c. Note that the overlap between both DFT models further supports sufficient data sampling in the experimental approach.

The DFT grid model also suggests that Pd-rich composition shows higher activity for ORR, with the optimal contents of Pd between 80–95% and 5–10% for both Ag and Au. These areas of optimal ORR activity in the DFT grid model partially overlapped with the experimental model. In addition, the top 2% most active compositions were plotted into a box plot in Fig. S14,† highlighting the elemental distribution trends and showing strong agreement in Pd-rich optima. However, two key discrepancies arise. In the DFT grid model, monometallic Pd exhibits lower ORR activity when compared to the trends in the experimental model. The reason for this discrepancy is not entirely clear at this point, however, two possible reasons can be suggested. First, the DFT calculations were performed for a surrogate 111-surface, the surface will have a perfect layer of oxygen and the adsorption energy of oxygen on Pd is suboptimal regarding the activity-expression we have defined. Structural effects may influence the results particularly for monometallic surfaces. Second, it should be noted that residual metal impurities are always present in the precursor salts, an effect that is not captured in the DFT calculations. Hence, a “real” Pd_100_Ag_0_Au_0_ data point is experimentally not feasible despite the use of “high grade” chemicals. In this context, it should be noted that while in the DFT model the predicted ORR activity of monometallic Pd is significantly lower than the optimal ORR activity, it quickly reaches close to optimal activity when Pd content at 80–95%.

The second discrepancy is the difference in “symmetry” of the ORR activity along the Pd–Au and Pd–Ag edges. The experimental model exhibits along the Pd–Au edge enhanced ORR activity as compared to the Pd–Ag edge, whereas the DFT model is more symmetrical along the two edges – at least in the region of high Pd content. This difference may be an experimental artifact, or it may indicate different segregation behavior of Au and Ag in the alloys. Supporting the artifact explanation is the observation from the SEM images (Fig. S15†) that the nanoparticles along the two edges exhibited morphological differences. Pd–Au nanoparticles displayed rough, cauliflower-like structures, suggesting that their ECSA was underestimated using the assumption of hemispherical nanoparticles for the active area determination. Pd–Ag nanoparticles by comparison appeared less “rough” and more “hemispherical”. In addition, it should be noted that the DFT model assumes a completely homogeneous alloy composition, whereas in reality, surface segregation can occur under ORR conditions. For example, literature reports suggest Ag in Pd–Ag alloys segregates to the surface reactive conditions, which could explain lower experimental activity than predicted by the DFT model.^41^ Hence, our approach might be suitable for indirectly detecting such phenomena occurring upon exposing electrocatalysts to a reactive environment.

## Conclusions

This study presents a robust framework for comparing experimental and DFT-derived composition–activity relationships. The proof of concept using a nanoparticle electrodeposition approach is applied for the Pd–Ag–Au trimetallic composition space and the ORR tested in alkaline conditions. Through a targeted sampling strategy and GPR modeling, we efficiently explored the ternary space using only 40 core compositions and 107 samples in total.

The key insights derived from this study include:

(1) Converging predictions: both experiments and DFT modeling identify Pd alloyed with small amounts of Ag and Au as the optimal catalyst composition.

(2) Discrepancies: DFT may underestimate Pd’s activity in nanoparticle-based electrocatalysts due to structural or compositional differences. While the experiments indicate an enhanced activity along the Pd–Au edge, this is not captured in the DFT calculations, most likely due to surface roughness and/or segregation effects.

Our methodology enables efficient exploration of multi-metallic and high entropy alloy electrocatalysts and can be extended to other systems, accelerating catalyst discovery and deepening understanding of composition–structure–activity relationships. Looking forward, our approach offers more than black-box model comparisons: it can flag deviations that hint at surface composition changes under reaction conditions. A major limitation in current catalyst screening is reliance on *ex situ*, bulk-averaged data. Our strategy helps pinpoint compositional regions where *operando* investigations are most warranted.

To advance our approach, the observed discrepancies between the experimental and the DFT modelling of the composition–activity relationship underscore the need for:

• Incorporating surface reconstruction, dynamic segregation, and stability effects into theoretical and experimental considerations.

• Experimental validation of even widely accepted computational predictions.

• In depth analysis of optimal compositions to capture the morphology of nanoparticle based electrocatalysts under operation conditions.

## Conflicts of interest

The authors declare no conflict of interest.

## Supplementary Material

FD-264-D5FD00095E-s001

FD-264-D5FD00095E-s002

## Data Availability

The raw data for this publication including electrodeposition files, SEM images, SEM-EDX data, ORR measurements, DFT calculation, and python scripts for data processing are available at Zenodo at https://doi.org/10.5281/zenodo.15577624.^40^
