# Supplementary material for: Electrochemical synthesis of high entropy nanoparticles and the exploration of the Pd–Ag–Au composition space for the oxygen reduction reaction
Source: Faraday Discuss. 2025 Jul 8;264:344–57. doi: 10.1039/d5fd00095e (PMC12495384; doi:10.1039/d5fd00095e)
Supplement: FD-264-D5FD00095E-s001 [file FD-264-D5FD00095E-s001.pdf]

Supplementary Information (SI) for Faraday Discussions.
This journal is © The Royal Society of Chemistry 2025

Supplementary Information

Electrochemical Synthesis of High Entropy Nanoparticles and the Exploration of the Pd-Ag-Au Composition Space for the Oxygen Reduction Reaction

Menglong Liu[1], Divyansh Gautam[1], Christian M. Clausen[2], Ahmad Tirmidzi[1], Gustav K.H. Wiberg[1], Jan Rossmeisl[2], and Matthias Arenz[1]*

[1] Department of Chemistry, Biochemistry and Pharmaceutical Sciences, University of Bern, Bern, 3012 (Switzerland)

[2] Center for High Entropy Alloy Catalysis (CHEAC) Department of Chemistry, University of Copenhagen, København Ø, 2100 (Denmark)

*matthias.arenz@unibe.ch

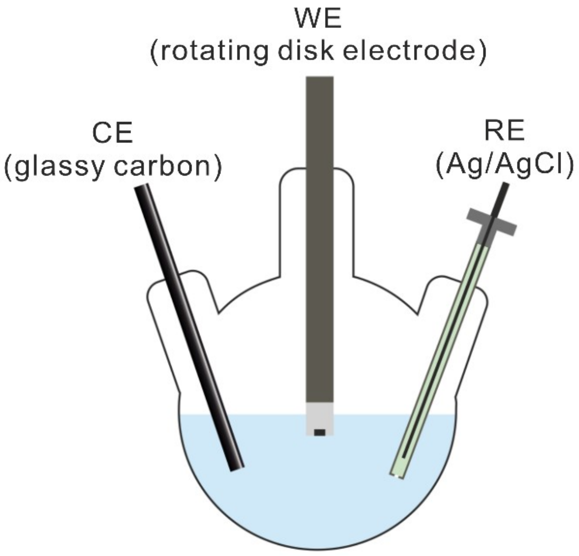

Figure S1. Schematic of the electrodeposition setup. A rotating disk electrode (RDE) is served as the working electrode (WE), a glassy carbon (GC) rod as the counter electrode (CE), and 3M Ag/AgCl electrode as the reference electrode (RE).

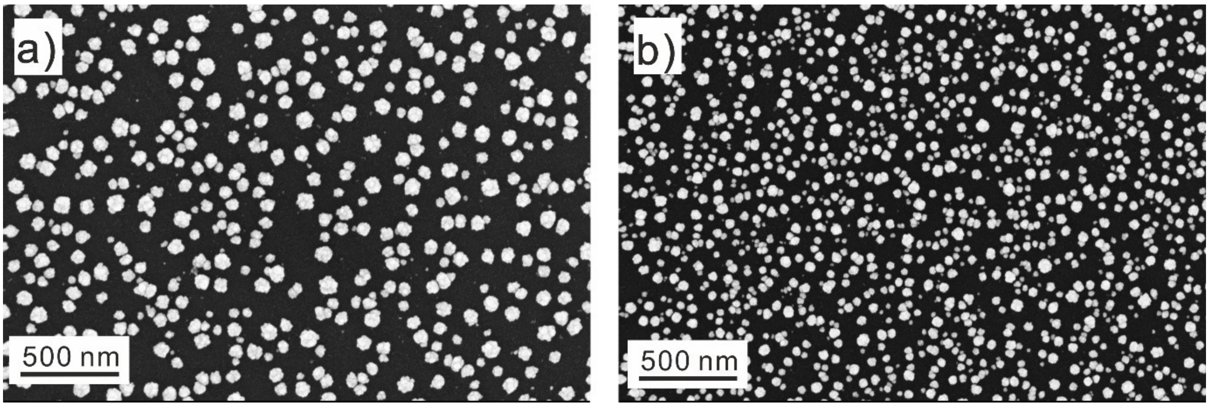

Figure S2. An example of tunning the particle size by altering the deposition current (nucleation rate). SEM images of the nanoparticles obtained with galvanostatic at current of (a) −70 μA for 60 s, and (b) −100 μA for 40 s, sample No.31. Due to the higher nucleation rate at larger absolute current, more particles with smaller size are obtained at current of −100 μA (1100 counts with average size of 52 nm with the same size of SEM image) compared to −70 μA (400 counts with average size of 85 nm). The particle size could also be tuned by varying the deposition potential or precursor concentration, which also resulting in different nucleation rate.

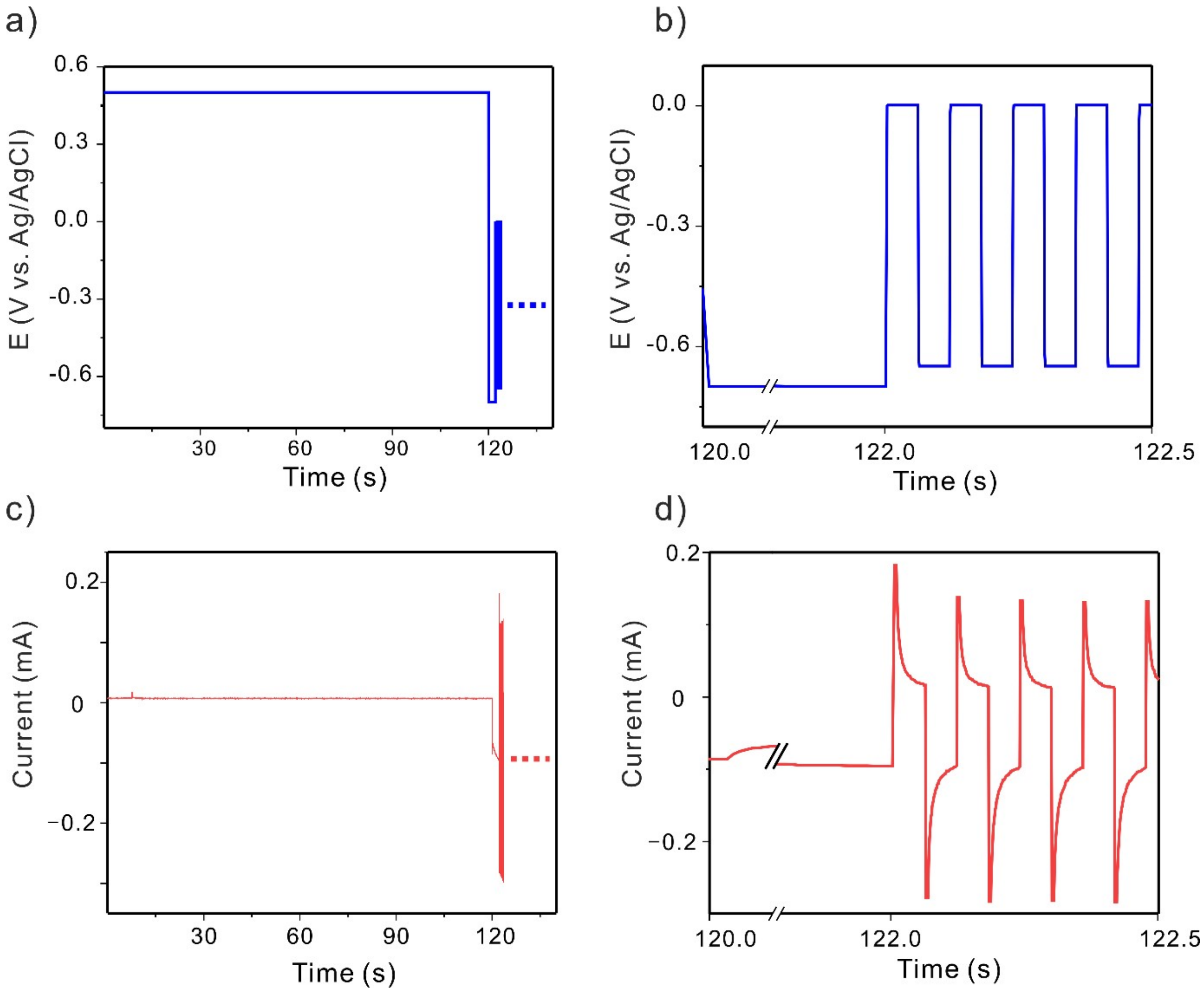

Figure S3. A representative potential and current data from pulse deposition, sample No.29. (a) The potential applied and (b) Zoom in from deposition time of 120 s to 122.5 s of (a); (c) The current and (d) Zoom in from deposition time of 120 s to 122.5 s of (c). The pulse deposition process includes three parts: in the first 120 s, relative a high potential of 0.5 V (vs Ag/AgCl, same for the potentials in deposition process) is applied to avoid any metal deposition. This is also indicated by the close to zero current; then a deposition potential of −0.7 V is applied for 2 s, aiming to form nuclei. Thereafter, the potential is alternated between −0.65 and 0 V with a frequency of 20 Hz each for a total time of 60-120 s.

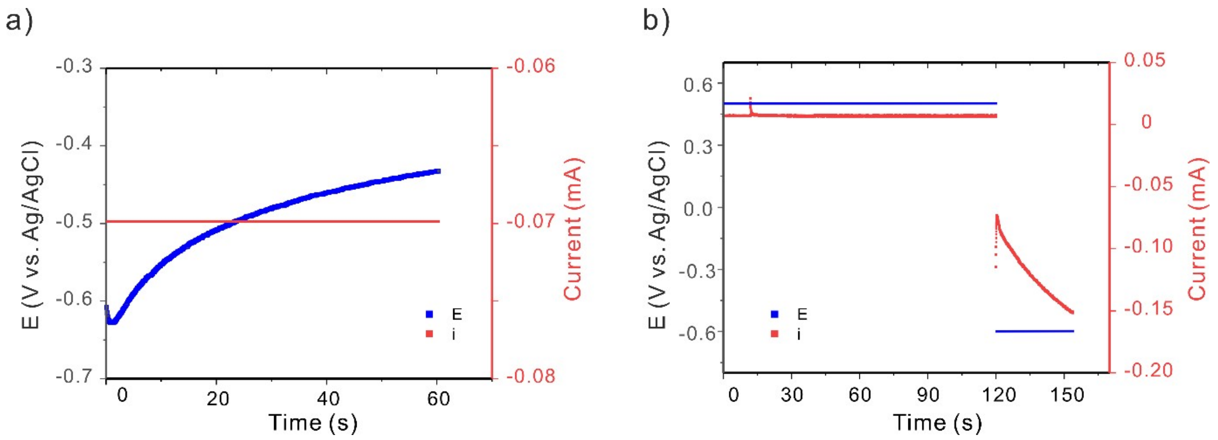

Figure S4. Representative potential and current data from (a) Galvanostatic deposition with a current of −70 µA recorded for sample No.30 and (b) Potentiostatic deposition at −0.6 V vs Ag/AgCl recorded for sample No.27. During galvanostatic deposition, the overpotential required to drive metal deposition shows a decreasing trend with time. This is because the metals are initially deposited on the glassy carbon surface; once nuclei are formed, subsequent deposition occurs on the metal surface, which requires less energy (overpotential). A similar trend is observed in potentiostatic deposition, as the absolute current continues to increase with time.

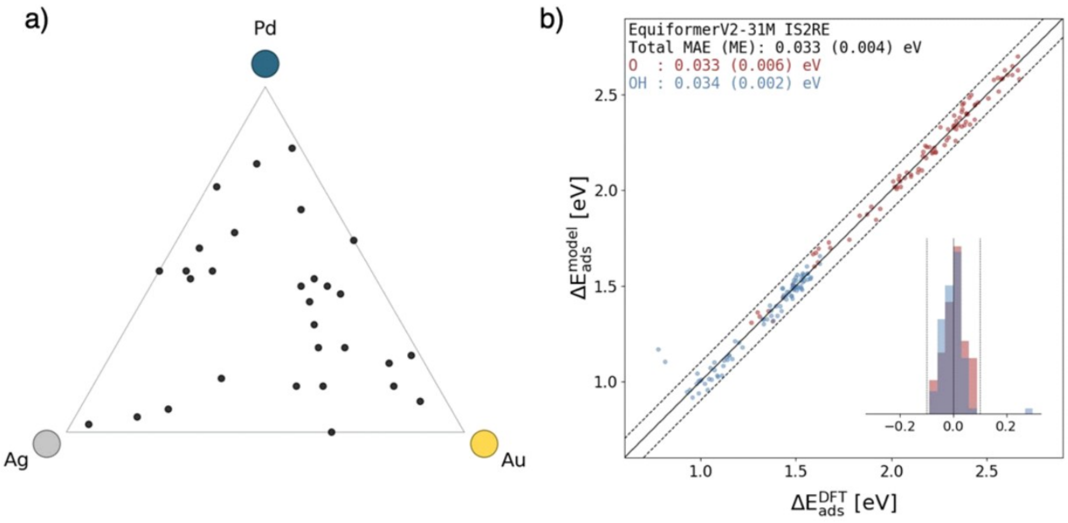

Figure S5. (a) Simplex of the Pd-Ag-Au composition space showing the compositions of the Pd-Ag-Au test set. The total number of unique slabs is 30 and the number of binding sites sampled are 176. (b) Parity plot showing prediction accuracy of the fine-tuned eqV2-31M model on the test set. Mean absolute error is shown for *O (red) and *OH (blue) separately as well as combined. The insert shows the distribution of errors as overlaid histograms for each species. The solid line represents perfect agreement between the model and the DFT calculated adsorption energy and the dashed lines represent ± 0.1 eV.

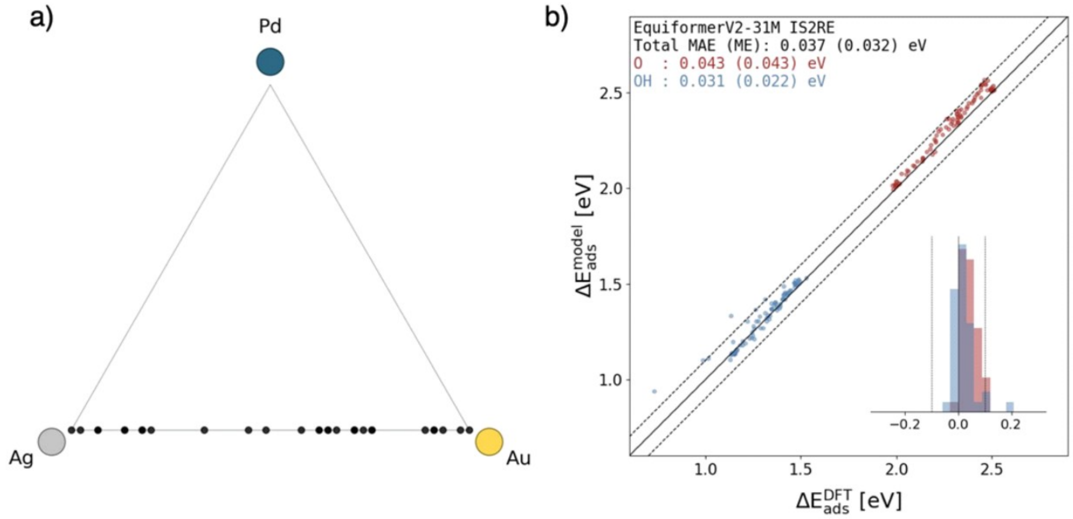

Figure S6. (a) Simplex of the Pd-Ag-Au composition space showing the compositions of the Ag-Au test set. The total number of unique slabs is 30 and the number of binding sites sampled are 177. (b) Parity plot showing prediction accuracy of the fine-tuned eqV2-31M model on the test set. Mean absolute error is shown for *O (red) and *OH (blue) separately as well as combined. The insert shows the distribution of errors as overlaid histograms for each species. The solid line represents perfect agreement between the model and the DFT calculated adsorption energy and the dashed lines represent ± 0.1 eV.

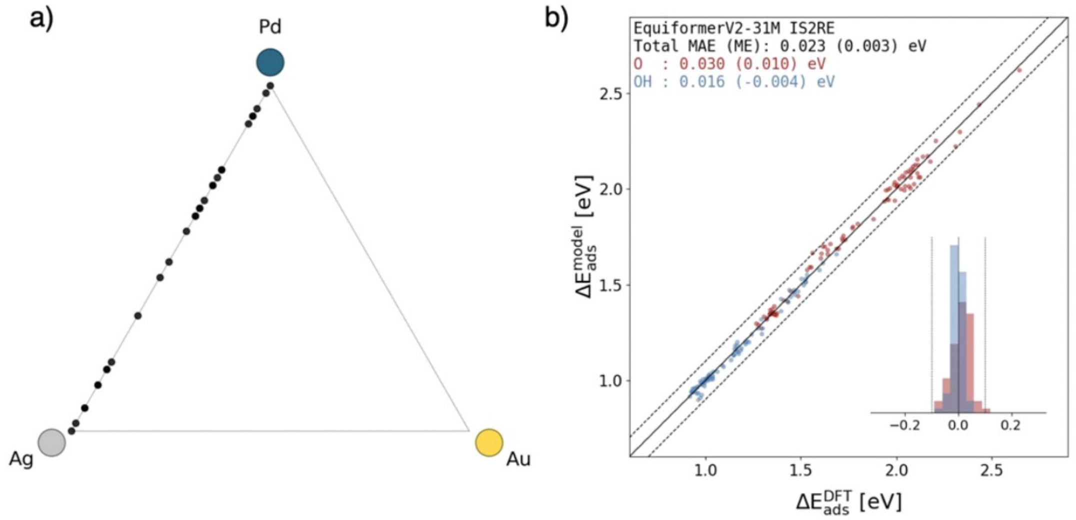

Figure S7. (a) Simplex of the Pd-Ag-Au composition space showing the compositions of the Ag-Pd test set. The total number of unique slabs is 30 and the number of binding sites sampled are 179. (b) Parity plot showing prediction accuracy of the fine-tuned eqV2-31M model on the test set. Mean absolute error is shown for *O (red) and *OH (blue) separately as well as combined. The insert shows the distribution of errors as overlaid histograms for each species. The solid line represents perfect agreement between the model and the DFT calculated adsorption energy and the dashed lines represent ± 0.1 eV.

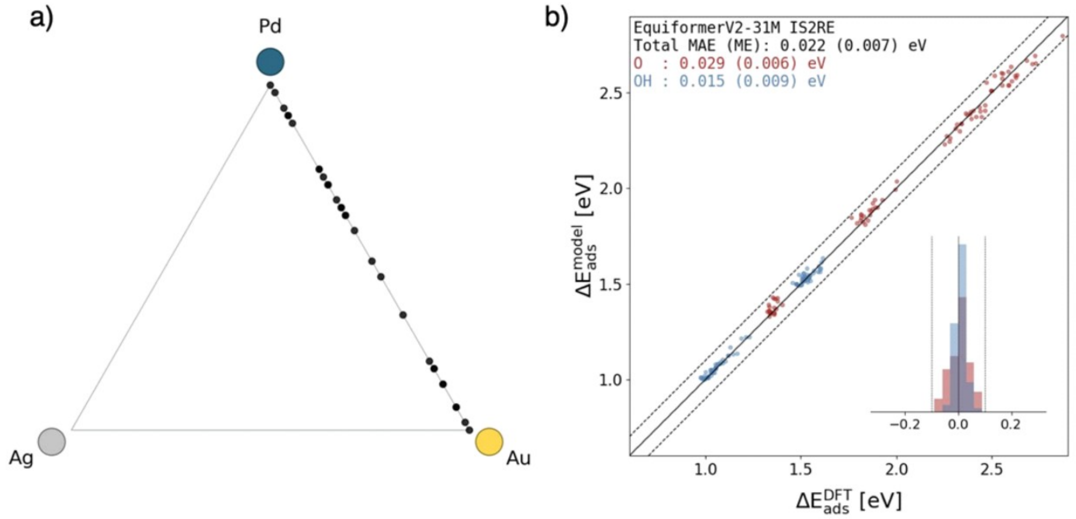

Figure S8. (a) Simplex of the Pd-Ag-Au composition space showing the compositions of the Au-Pd test set. The total number of unique slabs is 30 and the number of binding sites sampled are 180. (b) Parity plot showing prediction accuracy of the fine-tuned eqV2-31M model on the test set. Mean absolute error is shown for *O (red) and *OH (blue) separately as well as combined. The insert shows the distribution of errors as overlaid histograms for each species. The solid line represents perfect agreement between the model and the DFT calculated adsorption energy and the dashed lines represent ± 0.1 eV.

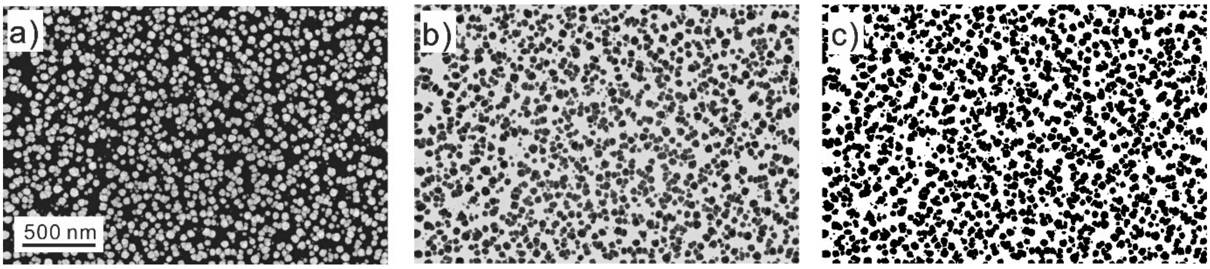

Figure S9. Procedure used by the Python script to determine particle coverage: (a) Original SEM image; (b) Inverted SEM image, converting nanoparticles from white to black and the background from black to white; (c) Contrast-enhanced image. Coverage is then calculated by determining the ratio of dark pixels to the total number of pixels in the image.

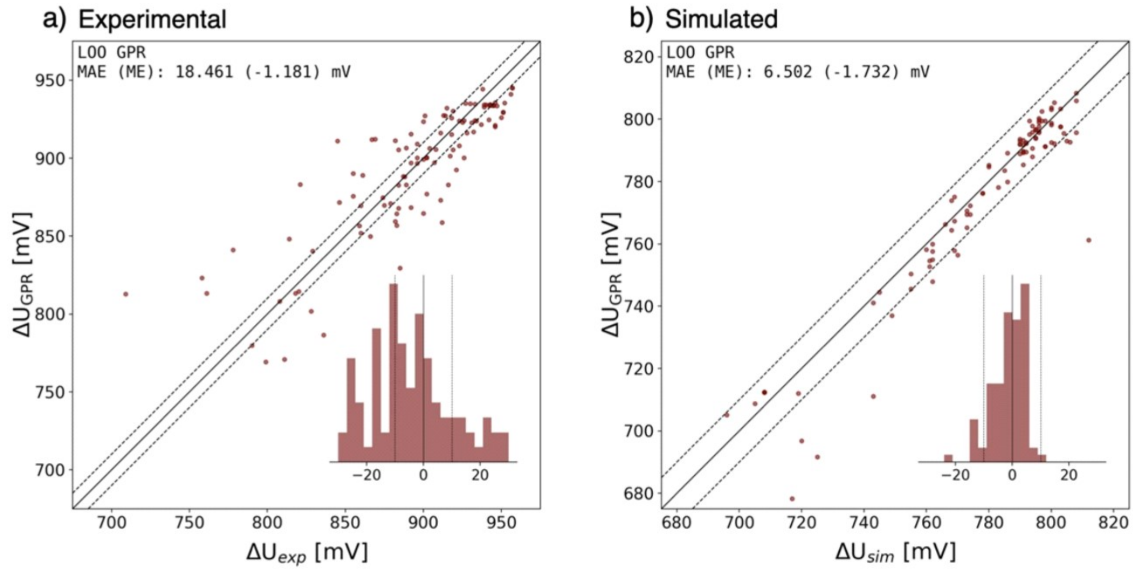

Figure S10. Parity plots showing the accuracy of the GPR model in a leave-one-out cross-validation procedure for (a) the RDE experimental measurements and (b) the simulated catalyst activities. The mean absolute error (MAE) is shown, and the mean error (ME) is shown in parenthesis. The insert shows a histogram with the distribution of errors. The solid line represents perfect agreement between the GPR model and the measured/simulated onset potential and the dashed lines represent ± 10 mV.

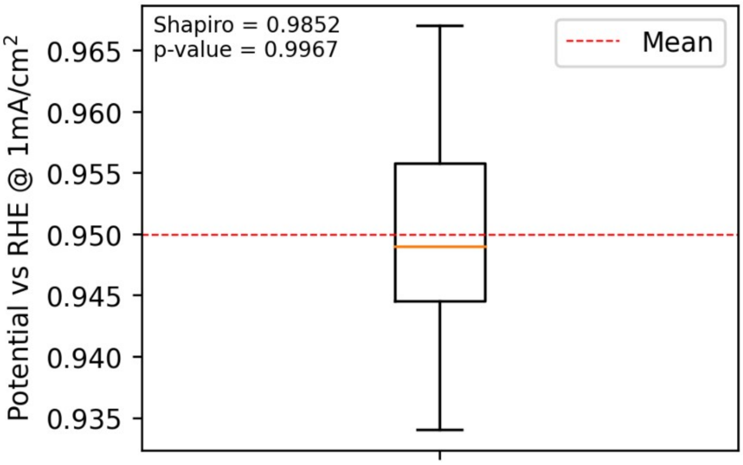

Figure S11. Box plot of the potential vs RHE at normalized current density of −1 mA/cm² from twelve repeated measurements. A targeted composition of $Pd_{77}Au_{23}$ was deposited twelve times, and the ORR activities of these samples were subsequently measured. The results yielded a mean value of 0.950 V and a median of 0.949 V, indicating strong agreement between central tendency metrics. The data followed a normal distribution (Shapiro-Wilk W = 0.9852, p = 0.9967) with low variance, as evidenced by the narrow interquartile range and absence of outliers. These findings demonstrate high experimental reproducibility and reliability of the measurement protocol. Note that these results are not included in the contour plot.

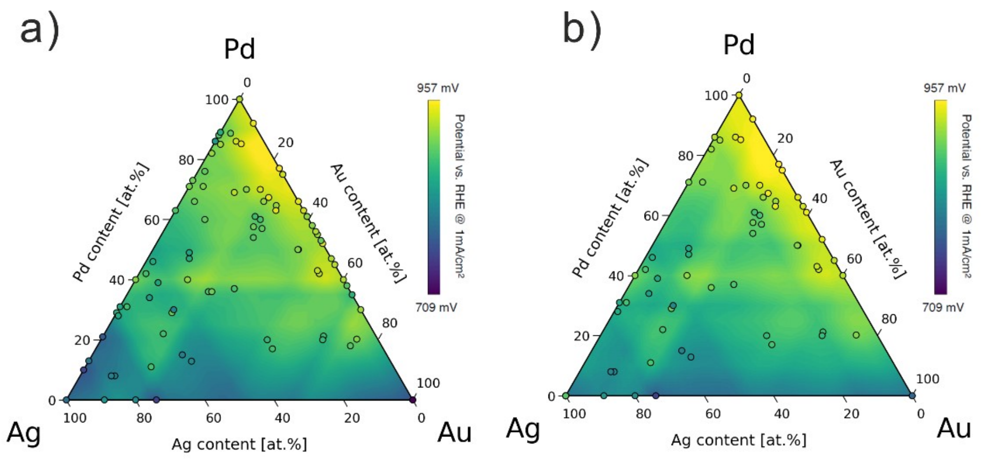

Figure S12. Experimental-based models with (a) all samples included and (b) potential outliers excluded. Potential outliers were identified based on observations of uneven nanoparticle deposition, surface contamination, or oxygen undersaturation. These potential outlier measurements are marked in the Excel file provided in the Supporting Information (SI).

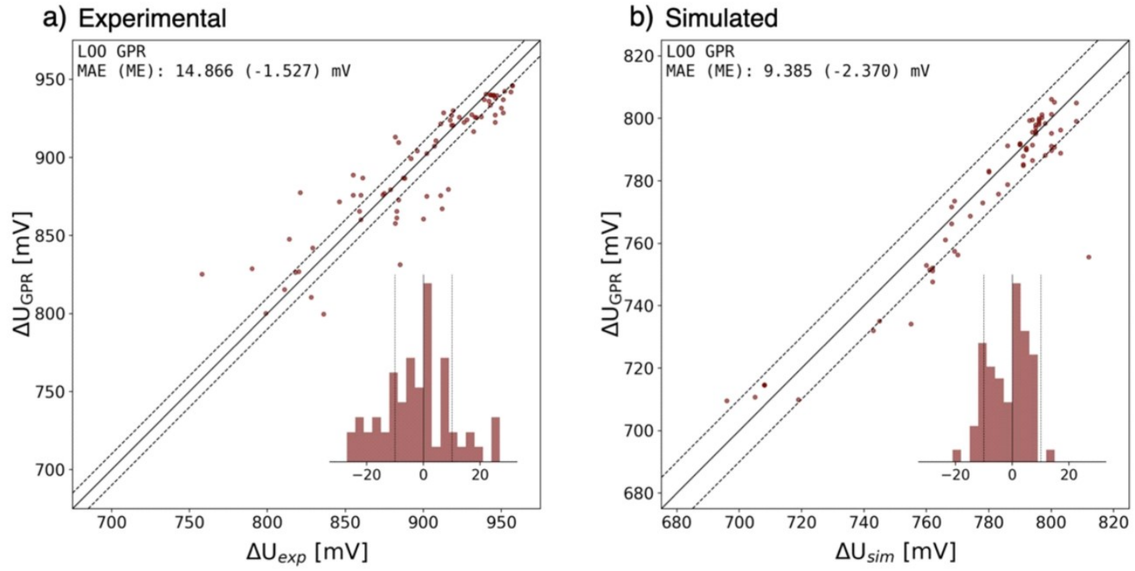

Figure S13. Parity plots showing the accuracy of the GPR model in a leave-one-out cross-validation procedure for (a) the RDE experimental measurements and (b) the simulated catalyst activities. Only the selected samples have been included. The mean absolute error (MAE) is shown, and the mean error (ME) is shown in parenthesis. The insert shows a histogram with the distribution of errors. The solid line represents perfect agreement between the GPR model and the measured/simulated onset potential and the dashed lines represent ± 10 mV.

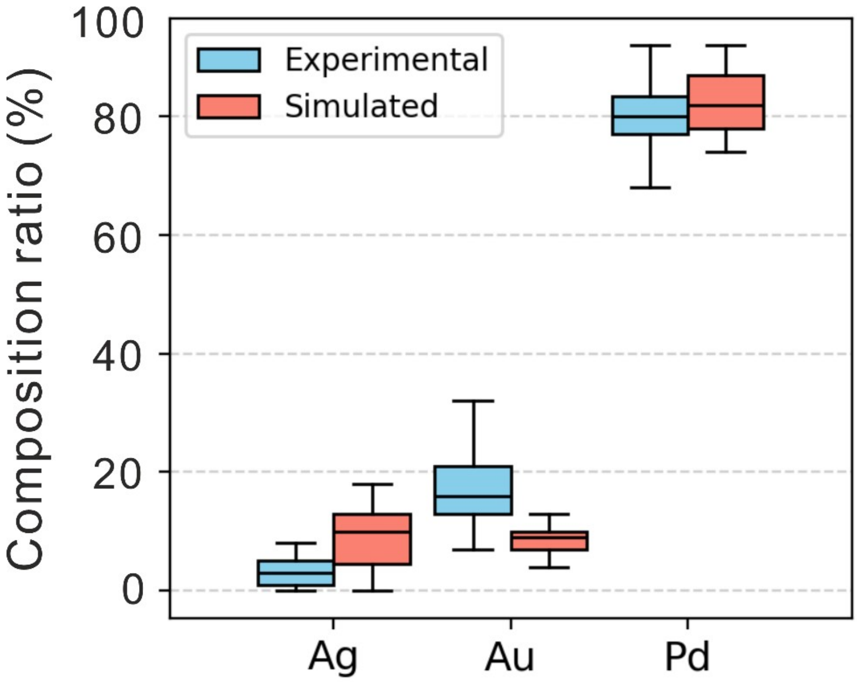

Figure S14. Comparison of the top 2% most active Pd-Ag-Au compositions identified from experimental and DFT-based models. This box plot provides a direct visual comparison of the most active compositions. In general, both models indicate that a high Pd content (>75 at.%) is critical for achieving high ORR activity. The experimental model predicts a broader optimal Pd range (70%–95%) compared to the DFT model (75%–90%). For Au, the experimental model shows a wider spread (8%–32%) than the DFT model (5%–15%). In contrast, Ag content exhibits a broader distribution in the DFT model than in the experimental results.

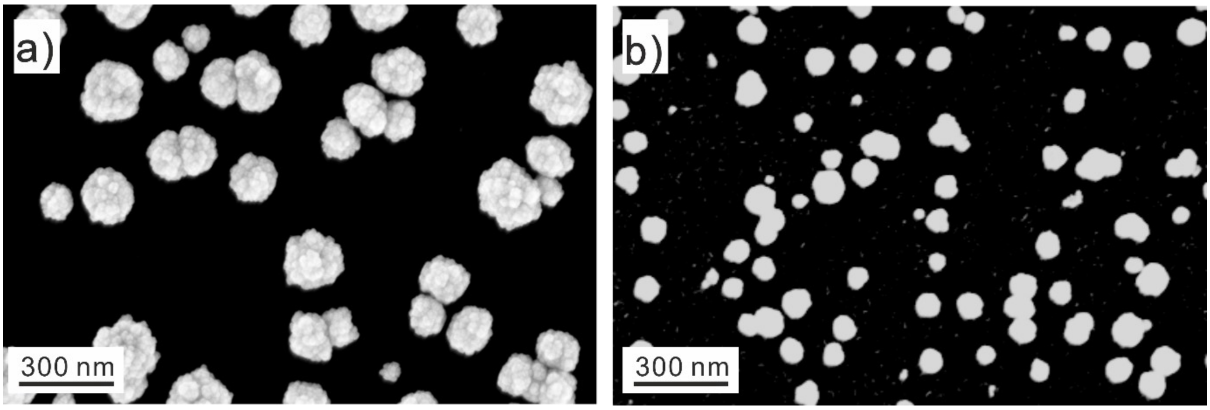

Figure S15. (a) Sample No.28 with a composition of $Pd_{63}Au_{37}$ exhibits particles with a cauliflower-like surface morphology. (b) Sample No.21 with composition of $Pd_{40}Ag_{60}$ shows particles that are relatively less rough and more “hemispherical”.
